# Frailty index, frailty phenotype and 6-year mortality trends in the FRASNET cohort

**DOI:** 10.3389/fmed.2024.1465066

**Published:** 2025-01-08

**Authors:** Sarah Damanti, Rebecca De Lorenzo, Lorena Citterio, Laura Zagato, Elena Brioni, Cristiano Magnaghi, Marco Simonini, Maria Pia Ruggiero, Simona Santoro, Eleonora Senini, Marco Messina, Francesca Farina, Costanza Festorazzi, Giordano Vitali, Paolo Manunta, Angelo Andrea Manfredi, Chiara Lanzani, Patrizia Rovere-Querini

**Affiliations:** ^1^Internal Medicine Unit, IRCCS San Raffaele Scientific Institute, Milan, Italy; ^2^Vita-Salute San Raffaele University, Milan, Italy; ^3^Nephrology and Dialysis Unit, IRCCS San Raffaele Scientific Institute, Milan, Italy; ^4^Scientific Technical Secretariat of the Ethics Committee. IRCCS San Raffaele Scientific Institute, Milan, Italy

**Keywords:** frailty index, frailty phenotype, mortality, community dwelling older people, older people

## Abstract

**Background:**

Frailty, a geriatric syndrome associated with adverse outcomes, lacks a universal definition. No consensus exists on the most effective frailty scale for predicting mortality.

**Methods:**

This prospective observational study followed community-dwelling volunteers for 6 years. Frailty was measured with the Frailty Index (FI) and the Frailty Phenotype (FP). Concordance was assessed using Cohen’s Kappa coefficients. Age-and sex-adjusted Cox regression analyses were conducted to evaluate the association with mortality.

**Results:**

Out of 1,114 participants (median age 72 years, IQR 69–77), 186 were classified as frail by the FI, 13 by the FP and 48 by both definitions. The concordance between the two measures was fair (*κ* = 0.26). Thirty-nine individuals died during the follow-up period. The FI showed a stronger association with mortality (HR 75.29, 95% CI 8.12–697.68, *p* < 0.001) compared to the FP (HR 3.3, 95% CI 1.45–7.51, *p* = 0.004). Individuals classified as frail by both definitions had the highest mortality risk and the highest FI scores (median 0.36).

**Conclusion:**

Definitions of frailty identify different individuals as frail. The FI was more closely related to mortality than the FP. Individuals classified as frail according to both definitions displayed the highest complexity (corresponding also ho higher FI scores) and the greatest mortality. The FI demonstrated a more accurate ability to predict mortality due to its comprehensive nature.

## 1 Introduction

### 1.1 Background

The aging population is growing. In 2021, one in ten people worldwide were aged 65 years old or older and it is expected that this age group will be 1 in 6 people globally in 2050 (United Nations) (1). The frail population is growing as well. Frailty is characterized by a decrease of physiological reserves and by a weakened response to stressors (2), but no unique definition still exists. As a consequence, its prevalence among older people varies between 4 and 59.1% depending on the screening tools used (3). This heterogeneity is due to the fact that each frailty definition captures specific aspects of this condition. Two main broad constructs applied in the clinical practice are the frailty phenotype (FP) (4), which describes frailty as a biological syndrome resulting from impairments in at least three out of five categories (global weakness, overall slowness, exhaustion, low physical activity and unintentional weight loss), and the frailty index (FI) (5), which consider frailty as an increased vulnerability to stressors deriving from the accumulation of health deficits in physical, psychological, cognitive and social domains. While the FP is centered on the loss of energy paradigm and on the physical dimension of frailty (6), the FI (5) captures cognitive, affective, social, physical and functional aspects of this geriatric syndrome in addition to comorbidities that can contribute to frailty.

Frail individuals are at an increased risk of poor clinical outcomes, including mortality (7). However, there is no consensus on which frailty definition best captures mortality risk. Few studies have directly compared the FI and the FP (8–13). Except for the study by Xue et al. (9) the FI has generally been found to be a stronger predictor of mortality compared to the FP. Moreover, Hamiduzzaman et al. (8) demonstrated that individuals classified as frail by both definitions had the highest mortality risk.

### 1.2 Objective

In this prospective observational study, we assessed the concordance between FI and FP and their ability to predict mortality over a six-year follow-up period in a well-characterized group of Italian community-dwelling older adults.

## 2 Methods

### 2.1 Study design

The Frailty and Sarcopenia Network (FRASNET) study was a prospective observational cohort study.

### 2.2 Setting

The study was performed at recreational centers, cultural centers, and retirement homes in the Milan and Monza Brianza regions, as well as at the San Raffaele Scientific Institute in Milan and the Cuggiono Hospital, located near Milan, Italy. The study received approval from the ethical board of the San Raffaele Scientific Institute (24/INT/2017). All participants provided written informed consent prior to their involvement in the study. Recruitment took place between April 1, 2017, and October 16, 2020. In 2023 the follow-up to assess patient mortality was conducted both through telephone interviews and by reviewing medical records.

### 2.3 Participants

The FRASNET study included both community-dwelling healthy volunteers and institutionalized patients (14). Inclusion criteria were: (i) age 65 years or older, (ii) ability to walk more than 500 meters without assistance, (iii) life expectancy of more than 6 months. Life expectancy was assessed by the enrolling physicians, based on their clinical judgment. Exclusion criteria were: (i) a cognitive impairment indicated by a Mini-Mental State Examination (MMSE) score < 18/30, (ii) inability to provide written informed consent, (iii) severe health issues (e.g., uncontrolled hypertension, recent fractures, myocardial infarction within the past year). Patients originally recruited from retirement homes (*n* = 19) and those missing data for the computation of frailty (*n* = 91) or for the analysis of body composition (*n* = 26) were excluded from the current analysis.

### 2.4 Variables, data sources, and measurements

Participants underwent comprehensive geriatric assessments (Supplementary Table S1), performed by a multidimensional équipe composed by physicians, nurses, physiotherapists and psychologists who received an *ad hoc* training for performing the scales and the evaluations of the study.

The comprehensive geriatric assessment included collecting demographic and psychosocial data through self-administered questionnaires, assessing comorbidities and medications, recording the number of falls and emergency department visits in the year preceding the evaluations, and taking anthropometric measurements (weight, height, waist circumference, body mass index (BMI)). Cognitive function was evaluated using the MMSE (15), mood with the 15-item Geriatric Depression Scale (GDS) (16), exhaustion with the Fatigue Severity Scale (FSS) (17), quality of life with the Short Form 36 (SF-36) Health Survey (18), and physical activity level with the Physical Activity Scale for the Elderly (PASE) questionnaire (19). Body composition was assessed using the Full Body Sensor Body Composition Monitor and Scale (OMRON), which employs bioelectrical impedance to estimate body composition. Muscle performance was evaluated using the Short Physical Performance Battery (SPPB) (20), with the chair-stand subtest serving as a measure of muscle strength (21).

Sarcopenia was defined according to the criteria of the European Working Group on Sarcopenia in Older People 2 (22); sarcopenic obesity according to the ESPEN and EASO criteria for (23). Specifically, sarcopenia was defined as a reduction in both muscle strength (chair test >15 s) and muscle mass (muscle mass < 32.9% in males and < 23.9% in females) (22), while obesity was identified by the presence of a fat mass ≥ 30% in men and ≥ 42% in women (23). In 2023 mortality was verified retrieved from the analysis of participants’ electronic health records and confirmed by a direct follow-up.

Frailty was measured though the FP and the FI. Since we often had no information on the weight in the year/months before the evaluations, we used a modified version of the FP which considered low BMI instead of unintentional weight loss as a criterium (24) (Supplementary Table S2). A 49-items FI was created by using the criteria defined by Theou et al. (25) (Supplementary Table S3). The 49 variables used to calculate the FI were obtained from the comprehensive geriatric assessment. Each deficit included in the FI was scored 0 for absence and 1 for presence. In cases of missing data, the FI was calculated using a reduced denominator, excluding missing items. Participants with more than 20% missing variables were excluded from FI computation. A cut-off of ≥0.25 was used to define frail individuals.

### 2.5 Bias

The prospective study could be affective by information bias related to modified frailty criteria. However, since the modified version of the frailty phenotype has been already applied in other studies (24) we thought that the risk of bias would have been greater by using an unreliable reported weight loss. An attrition bias could have been related to participants lost to follow-up. However, we checked though electronic medical records information related to mortality also for patients lost to follow-up.

### 2.6 Study size

The sample size of the FRASNET study of at least 1,198 participants has been calculated with a two-sided t-test with an alpha of 5% and a power of 80% by assuming that the mutated allele for the Klotho gene between the frail and non-frail group was (0.12 in frail individuals and 0.19 in non-frail people).

### 2.7 Quantitative variables and statistical methods

Descriptive statistics were used to show the baseline characteristics of the study population. Continuous variables were presented as mean and standard deviations (SD), when normally distributed, or with median and interquartile range (IQR), when data had a skewed distribution. Dichotomous variables were presented as number (N) and percentage (%). Kappa Cohen coefficients were used to assess the concordance among the different frailty definitions. The differences of distribution of continuous and categorical variables among the different frailty categories were computed with the Kruskal-Wallis’s and the Chi-squared tests, respectively. The Chi-squared test was also used to assess the difference in mortality between frail and non-frail individuals. Cox regression models adjusted for age and sex were used to assess the association between frailty and mortality. Analyses were performed first considering FI as a continue variable and the FP as a 6-level ordinal variable (ranging from 0 to 5). Then we applied dichotomous categorical formats of frailty classifying individuals as frail according to a FI ≥ 0.25 and FP ≥ 3 and non-frail when they had FI < 0.25 and FP < 3.

All statistical analyses were performed with SPSS version 25.0 (SPSS Inc., Chicago, IL, United States).

## 3 Results

### 3.1 Participants and descriptive data

**Figure 1:**
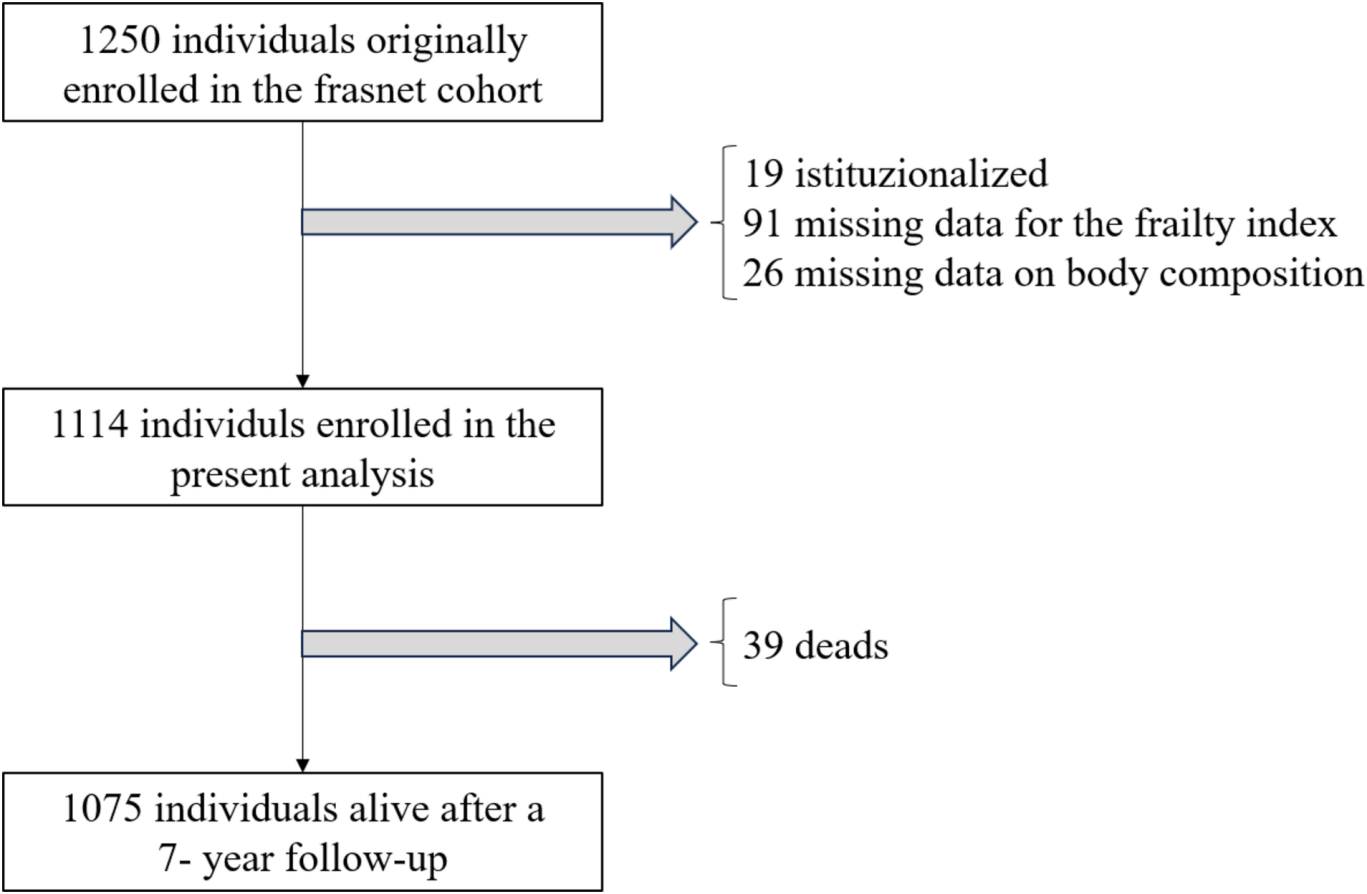
Flow chart of the FRASNET study recruitment and follow-up.

Out of the 1,250 participants enrolled in the FRASNET study, 1,114 were considered for this analysis. Exclusions comprised 19 institutionalized patients, 91 individuals with more than 20% missing data for the Frailty Index calculation, and 26 participants with missing body composition data (Figure 1). The study sample had a median age of 72 years (lowest age 65 years, highest age 93 years) and was composed for the 60.5% by females. Table 1 illustrates participant main characteristics and Supplementary Table S4 the main characteristics according to their frailty status.

**Table 1:** Main characteristics of the study population.

	All (*N* = 1,114)
Age	72 (IQR 69–77)
Females	674 (60.5%)
Marital status	
Married	788 (70.7%)
Widower/divorced	270 (24.2%)
Single	56 (5%)
Smoke	382 (34.3%)
Economic status	
< 10.000 euro/year	97 (8.7%)
> 10.000 euro/year	1,000 (89.8%)
Education	
Primary School	158 (14.2%)
Middle school	265 (23.8%)
High school	488 (43.8%)
University	200 (18%)
Weight (kg)	69.5 (IQR 61.6–78.7)
Waist circumference (cm)	92 (IQR 85–101)
BMI (kg/m2)	26.8 (IQR 24–29.4)
SPPB	11 (IQR 9–11)
Gait speed (m/s)	1.16 (IQR 1.0–1.31)
PASE	102 (IQR 65–152)
Chair test (sec)	13.0 (IQR 11.0–15.7)
Muscle mass (%)	27.9 (IQR 24.9–31.5)
Fat mass (%)	33 (IQR 26.2–40.1)
Visceral fat (%)	11 (IQR 8–14)
Sarcopenic non obese	51 (4.6%)
Sarcopenic obese	86 (7.7%)
FI	0.11 (IQR 0.07–0.20)
MMSE	27 (IQR 26–30)
Fatigue Severity Scale	26 (IQR 16.5–36)
Hypertension	670 (60.1%)
Diabetes	109 (9.8%)
Dyslipidaemia	155 (13.9%)
Chronic Kidney Disease (i.e., GFR < 60 mL/min)	221 (19.8%)
Cardiovascular incidences	217 (19.5%)
Previous stroke	56 (5%)
Psychiatric incidences	101 (9.1%)
Any fall in the year previous the evaluation	243 (21.8%)

BMI, Body Mass Index, GFR, Glomerula Filtration Rate, PASE, Physical Activity Scale for the Elderly, SPPB, Short Physical Performance Battery.

### 3.2 Outcome data

**Figure 2:**
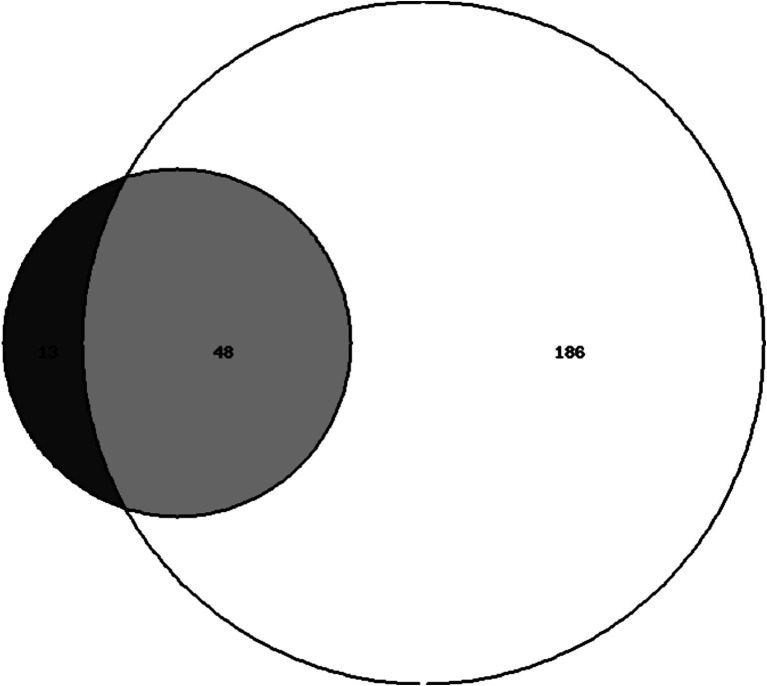
Overlap between the frailty index (FI) ≥ 0.25 and the frailty phenotype definitions in the FRASNET cohort. Dark gray circle = 13 individuals frail according to the frailty phenotype. Light gray circle = 48 individuals frail according to both definitions. White circle = 186 individuals frail according to the frailty index cut off ≥ 0.25.

The median FI score was 0.11 (IQR 0.07–0.20). According to the FI, 234 individuals (21%) were classified as frail, whereas 61 individuals (5.5%) were classified as frail according to the FP. Among these, 48 individuals met both frailty definitions, while 186 were considered frail only by the FI definition, and 13 were considered frail only by the FP definition (Figure 2). Individuals classified as frail according to both frailty definitions were mainly females (77.1%) and showed the lowest levels of education (45.8% primary school), income (19.6% < 10.000 euros/year), physical (median SPPB 7, median chair test 19.2 s) and cognitive performance (median MMSE 26). They also had the highest prevalence of fatigue (median FSS 48.5), sarcopenia (8.3%), sarcopenic obesity (41.7%), polypharmacy (54.2%), Emergency Department visits (34%), and falls (45.7%) in the year preceding the study evaluations (Table 1). Participants classified as frail according to the FI were older compared to robust people (median age 76 vs. 71, *p* < 0.001), with a higher percentage of females (69.9% vs. 57.7%, *p* < 0.001), sarcopenic obesity (41.7% vs. 5.5%, *p* < 0.001) polypharmacy (50% vs. 18%, *p* < 0.001), fatigue (median FSS 33 vs. 22, *p* < 0.001), falls (27.7% vs. 29.9%, *p* < 0.001) and emergency department accesses (28.8% vs. 19.1%, *p* < 0.001) in the year preceding the evaluation and a lower level of education (primary school 24.7% vs. 9.6%, *p* < 0.001) and physical activity (median PASE 71 vs. 112, *p* < 0.001) (Table 1). Also individuals classified as frail according to the FP were older compared to robust ones (median age 76 vs. 71 years, *p* < 0.001), with a lower level of education (primary school 53.8% vs. 9.6%, *p* < 0.001), physical activity (median PASE 39 vs. 112, *p* < 0.001) and physical performance (median SPPB 9 vs. 11, *p* < 0.001; median chair test 16.5 vs. 12.5, *p* < 0.001), a higher BMI (29 vs. 26.6, *p* < 0.001), fatigue (median FSS 39 vs. 22, *p* < 0.001) and prevalence of sarcopenia (30.8% vs. 3.9%, *p* < 0.001) and sarcopenic obesity (38.5 vs. 5.5%, *p* < 0.001), more falls (30.8% vs. 19.1%, *p* < 0.001) and ED accesses (30.8% vs. 19.9%, *p* < 0.001) in the year preceding the evaluations (Table 1). The concordance between the FI and FP (*k* = 0.26, *p* < 0.001) was only fair.

### 3.3 Main results

**Figure 3:**
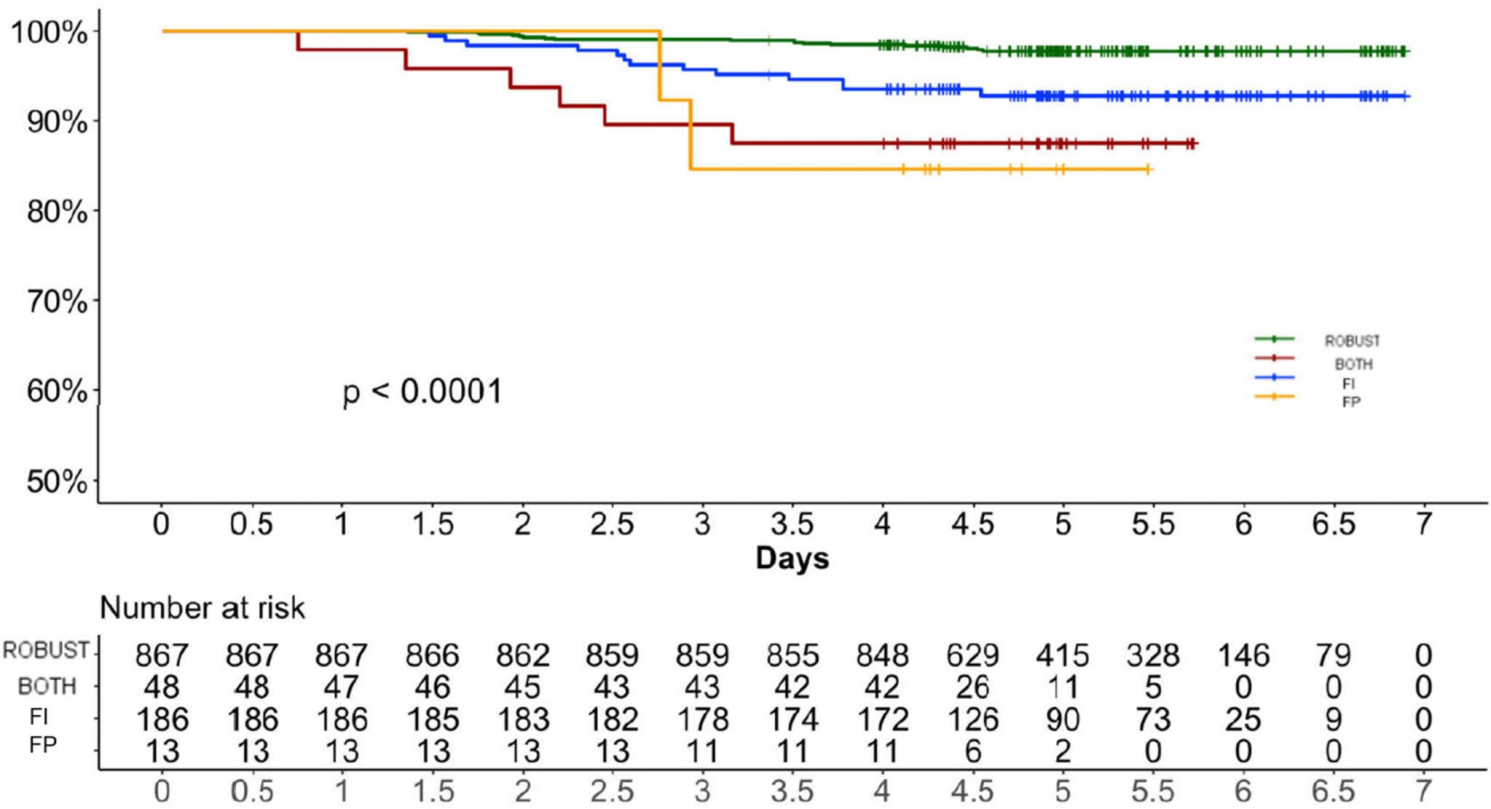
Age and sex-adjusted cox regressions showing the association between frailty and mortality. FI Frailty Index. FP Frailty Phenotype.

Between the enrolment in 2017 and the 2023 follow-up, 39 individuals (3.5%) had died. People who died were older and frailer (median age 78, IQR 73–84; median FI 0.23, IQR 0.11–0.34) than survivals (median age 72, IQR 69–76; median FI 0.11, IQR 0.07–0.20). Mortality was 2.1% in robust individuals and 8.5% in frail individuals (*p* < 0.001). The age- and sex-adjusted Cox regression models confirmed that people who were frail had a significantly higher mortality compared to robust individuals (Figure 3). FI had a stronger association with mortality (HR 75.29, 95% C.I. 8.12–697.68, *p* < 0.001) compared to the FP (HR 1.89, 95% C.I. 1.40–2.55, p < 0.001) when these variables were considered as continuous and ordinal, respectively. Individuals who were frail according to both frailty definitions displayed the highest mortality risk (HR 5.26, 95% C.I. 1.94–14.22, *p* = 0.001) compared to individual classified frail according to the FI (HR 2.83, 95% C.I. 1.32–6.05, *p* = 0.007) and the FP (HR 4.71, 95% C.I. 1.05–21.13, *p* = 0.043) displayed the highest median frailty index score (Table 1).

## 4 Discussion

### 4.1 Key results

In this observational study we found that the prevalence of frailty in a sample of geriatric Italian community dwelling volunteers was higher when computed with FI than with the FP. The concordance between FI and FP was only fair. Both FI and FP predicted mortality, but FI displayed a stronger association with mortality than the FP. People who were frail according to both definitions had the highest mortality, but had also the highest median FI score.

### 4.2 Interpretation

Our findings regarding the prevalence of frailty, with rates of 21% using the FI and 5.5% using the FP, align closely with those reported in a European population study (SHARE) which aimed at assessing the effects of health, social, economic and environmental policies over the life-course (frailty in the SHARE study: 21.6% with the FI and 11% with the FP) (26). Our data were also in line with the results of an American population (NHANES) study designed to assess the health and nutritional status United States citizens (frailty in NHANES 22% with the FI and 6% with the FP) (27). Finally, our detections on frailty in ambulatory patients closely align with those of a recent meta-analysis on frailty prevalence in community-dwelling older individuals worldwide (frailty prevalence in Europe: 22% with the FI and 8% with the FP) (28). The poor concordance (*k* = 0.26) between the FI and FP reflects the different nature of these instruments (29). It would be inappropriate to view the FP and the FI as interchangeable or equivalent tools. These two instruments serve different purposes and should be regarded as complementary. The FP is based on a predefined set of criteria that assess the presence or absence of specific signs or symptoms. Therefore, it can be utilized during the initial interaction with a subject and does not require prior clinical evaluation, making it useful as a screening tool for initial risk stratification across various profiles. Since the FP focuses on broad signs or symptoms, it primarily serves as a warning for potential issues. In contrast, the FI cannot be easily applied during the first contact with a patient, as it is derived from a comprehensive geriatric assessment. Anyway, once this assessment is complete, the FI provides valuable information for ongoing monitoring, and it is more sensitive to changes than the categorical frailty phenotype. Furthermore, because the FI is largely based on clinically classified conditions, it reflects a risk profile that may align more closely with the clinician’s assessment, potentially identifying vulnerabilities that differ from those indicated by the frailty phenotype (29).

Our finding on the poor concordance between the FP and FI was also previously highlighted in the NHANES study (*k* = 0.166) (27). The FP is a screening instrument that is easily applicable in clinical evaluations. However, it focuses solely on the physical dimension of frailty and may not capture the cognitive, social, and psychological aspects of frailty, nor the related comorbidity burden. This limitation could explain the weaker association of FP with mortality compared to the FI.

Frailty has been extensively associated with mortality (11, 30, 31). Prior systematic reviews indicated that the FI tool might be more effective than FP in predicting overall mortality (32–35) and that using continuous and ordinal formats instead of categorical ones in either tool improved their capacity to forecast overall mortality (13). Our findings are in line with the literature and in our work the FI considered as continuous variable displayed the strongest association with mortality.

Our study has the merit of having compared the risk of mortality in mutually exclusively categories of frailty (i.e., frail according only to the FI, frail according only to the FP and frail according to both definitions) in community dwelling individuals. Consistent with the findings of Hamiduzzaman et al. (8), we showed that individuals classified as frail according to both frailty definitions exhibited the highest mortality risk. However, key differences exist in the populations studied and our findings are novel. Hamiduzzaman’s research focused on American dialysis-dependent patients, comparing the Veterans Affairs Frailty Index (VAFI) with the FP. They found poor concordance between the two tools but noted that frailty, regardless of the measure, predicted higher mortality within this specific population. Notably, individuals classified as frail by both definitions showed the highest mortality risk compared to those deemed robust. However, their study did not directly compare mortality risk across mutually exclusive categories of frailty—those who were frail according to FI alone, FP alone, or both—something our study does. Moreover, our research centered on a relatively healthy, community-dwelling cohort, rather than dialysis patients. Additionally, our study provides novel insights into the interaction between sarcopenia, sarcopenic obesity, and frailty in relation to mortality, especially among individuals classified as frail by both FI and FP. This subgroup was the most compromised in the study, characterized by the highest FI scores, the lowest levels of education, income, physical and cognitive performance. They also had the highest prevalence of fatigue, polypharmacy, Emergency Department visits, and falls in the year preceding the study evaluations. It is important to underline that sarcopenia and sarcopenic obesity, which were highly prevalent in this subgroup (8.3 and 41.7%, respectively), may have contributed to the increased mortality. Both conditions have been associated with an elevated risk of mortality (36). It is interestingly to underline that in our sample mortality was particularly low (only 3.5%), even considering the intercurrent COVID-19 pandemics, when all cause excess deaths reached nearly the 60% in the North regions of Italy (37). Indeed, the study population was constituted mainly by robust and active individuals and could be seen as a model of healthy ageing.

To sum up, our study confirmed that the FI is a superior tool for predicting mortality in community dwelling older adults when compared to the FP. This finding is consistent with previous studies that have shown the FI’s greater predictive power for mortality due to its ability to capture a wider range of health deficits, including physical, cognitive, and social factors, as well as comorbidities (33, 34) which can contribute to mortality risk.

### 4.3 Limitations

Anyway, there are some limitations that should be considered in our study. We assessed two out of numerous indices currently used to evaluate frailty the FP and FI selected based on the strong predictive capabilities demonstrated in other studies (38, 39). Inclusion of other frailty scales would have given a more comprehensive evaluation of the association between frailty and mortality. Additionally, while we analyzed a substantial prospective cohort of community-dwelling older adults, our study was regionally focused, and the generalizability of our findings should be validated through future multicenter studies that include populations beyond just ambulatory patients.

Self-reported data for psychosocial variables and physical activity may be prone to recall bias or social desirability bias, potentially affecting the accuracy of some assessments.

### 4.4 Generalisability

Increasing awareness of the prevalence of frailty and its associated mortality risk is a crucial first step in promoting the adoption of preventive and therapeutic measures to mitigate the negative consequences of frailty.

## 5 Conclusion

Frailty is a common geriatric condition found in up to one-fifth of Italian community dwelling older adults. Frailty is closely linked to mortality. Various definitions of frailty identify different individuals as frail. The FI, due to its comprehensive nature showed a stronger association with mortality. Individuals classified as frail by both frailty definitions were the most compromised, displayed the highest FI score and the faced the highest mortality risk.

## Data Availability

The raw data supporting the conclusions of this article will be made available by the authors, without undue reservation.
